# Transcranial direct current stimulation combined with exercise therapy for chronic low back pain: a systematic review and meta-analysis

**DOI:** 10.3389/fnhum.2025.1721182

**Published:** 2026-01-16

**Authors:** Rui Xia, Shihan Lu, Lunan Zhao

**Affiliations:** 1 College of Sports Science, Qufu Normal University, Qufu, China; 2 School of Physical Education and Sports Science, South China Normal University, Guangzhou, China

**Keywords:** transcranial direct current stimulation, exercise therapy, chronic low back pain, add-on effect, meta-analysis

## Abstract

**Background:**

Chronic low-back pain (CLBP) is a leading cause of disability, with current treatments offering only modest benefits. Transcranial direct-current stimulation (tDCS) may enhance exercise therapy by modulating cortical excitability and pain-inhibitory pathways. This systematic review and meta-analysis quantified the additive effect of combining tDCS with structured exercise in adults with CLBP.

**Methods:**

We searched PubMed, Web of Science, CENTRAL, Embase, and CNKI up to 25 September 2025. Randomized controlled trials (RCTs) comparing active tDCS plus identical exercise therapy vs. sham tDCS plus the same exercise in adults with CLBP (≥ 12 weeks) were included. Risk of bias was assessed using Cochrane RoB 2.0. Weighted mean difference (WMD) and standardized mean difference (SMD) were calculated for pain and function, respectively.

GRADE was used to assess certainty.

**Results:**

Five RCTs (*n* = 195) were included. For pain intensity (4 studies, *n* = 173), tDCS showed a significant additive effect (WMD = −0.99, 95% CI: −1.68 to −0.31, *p* = 0.006, *I*^2^ = 60.1%). For physical function (five studies, *n* = 195), the effect was favorable but non-significant (SMD = −0.65, 95% CI: −1.87 to 0.57, *p* = 0.28, *I*^2^ = 90.7%). Meta-regression indicated intervention duration significantly moderated functional outcomes (β = 0.56, *p* < 0.001). GRADE certainty was moderate for pain and low for function.

**Conclusion:**

Anodal tDCS combined with exercise provides a modest but significant additional reduction in pain intensity for CLBP. Longer intervention duration may enhance functional outcomes. Clinical significance should be interpreted cautiously. Larger, well-designed trials are needed to confirm these findings and optimize stimulation parameters.

**Systematic review registration:**

https://www.crd.york.ac.uk/PROSPERO/view/CRD420251151315, identifier CRD420251151315.

## 1 Introduction

Chronic low-back pain (CLBP)—defined as pain localized between the costal margin and the gluteal fold, with or without leg pain, persisting for ≥ 12 weeks in the absence of identifiable specific structural pathology—is among the leading causes of years lived with disability worldwide (Balagué et al., 2012; GBD 2019 Diseases and Injuries Collaborators, 2020). Despite extensive research into therapeutic interventions, currently available evidence-based treatments confer only modest benefit; exercise therapy, recommended as first-line management by major clinical guidelines, yields an average pain reduction of merely 1.2–1.8 units on a 0–10 visual analog scale (National Guideline Centre, 2016; Qaseem et al., 2020; Hayden et al., 2021). This ceiling effect has prompted investigation of adjunctive interventions that might amplify therapeutic outcomes through synergistic mechanisms. Emerging evidence implicates central pain mechanisms in the pathophysiology of CLBP, including altered excitability of the primary motor cortex (M1) and dorsolateral prefrontal cortex, impaired descending pain-inhibitory pathways, and aberrant connectivity within cortico-thalamo-spinal networks (Apkarian et al., 2011; Seminowicz et al., 2011; Kregel et al., 2015). These observations provide a neurobiological rationale for non-invasive brain stimulation as a therapeutic adjunct. Transcranial direct-current stimulation (tDCS) delivers weak direct current (1–2 mA) to targeted cortical regions, producing lasting modulation of cortical excitability via mechanisms such as potentiation of glutamatergic transmission and activation of descending pain-inhibitory pathways (Nitsche and Paulus, 2000; DaSilva et al., 2011). Animal studies demonstrate that anodal tDCS increases glutamatergic synaptic transmission in M1 and activates the periaqueductal gray–anterior olivary–dorsolateral funiculus spinal pathway, significantly reducing hyperexcitability of dorsal-horn neurons (Bolzoni et al., 2013). In human pain models, single-session anodal high-definition tDCS over M1 increases cold-pain thresholds in healthy individuals compared with sham, indicating effective modulation of the sensory-discriminative dimension of pain perception (Li et al., 2022). Clinical studies in CLBP populations show that tDCS alone provides short-term pain relief and improved trunk motor control (Straudi et al., 2018; Ahn et al., 2019; Corti et al., 2022).

The rationale for combining tDCS with exercise rests on complementary mechanisms: tDCS-induced cortical priming may enhance motor-learning and neuroplastic responses to structured exercise, while exercise provides sensorimotor input that consolidates tDCS-induced cortical changes. However, existing evidence is limited by small sample sizes (often < 30 participants), heterogeneous stimulation parameters, and inadequate control conditions that preclude isolation of the additive effect of tDCS from that of exercise. Studies of tDCS in CLBP exhibit marked variability in protocols and outcomes, yielding mixed findings regarding its clinical efficacy (Ibrahim et al., 2025). Differences in treatment protocols, study design, sample characteristics, and outcome measures likely contribute to these inconsistencies. Consequently, current clinical guidelines have not endorsed tDCS as an adjunctive therapy owing to lack of high-quality evidence (National Guideline Centre, 2016; Qaseem et al., 2020). We therefore undertook this systematic review and meta-analysis to quantify the net additive benefit of tDCS when superimposed on an identical exercise program, specifically addressing the research question: In adults with chronic non-specific low-back pain, does anodal tDCS combined with structured exercise therapy produce clinically meaningful incremental improvements in pain intensity and physical function compared with sham tDCS delivered alongside the identical exercise program?

## 2 Methods

This systematic review was conducted in accordance with the 2020 Preferred Reporting Items for Systematic Reviews and Meta-Analyses (PRISMA) guidelines (Page et al., 2021) and was prospectively registered with the PROSPERO database before the literature search (registration number: CRD420251151315).

### 2.1 Literature search

Five electronic databases were searched from inception to 25 September 2025:

PubMed/MEDLINE, Web of Science, the Cochrane Central Register of Controlled Trials (CENTRAL), Embase, and the China National Knowledge Infrastructure (CNKI). The search strategy combined Medical Subject Headings (MeSH) and free-text terms related to chronic low-back pain (CLBP), transcranial direct-current stimulation (tDCS), and exercise therapy (Supplementary material). Languages were restricted to Chinese and English. Reference lists of included studies and relevant systematic reviews were manually screened for additional eligible trials.

### 2.2 Literature screen and data extraction

After duplicate removal in EndNote X9, three reviewers independently screened titles, abstracts, and full texts for eligibility; disagreements were resolved through discussion or consultation with a fourth reviewer. Two reviewers independently extracted data using a standardized form, capturing study characteristics, participant demographics, intervention details (tDCS parameters, exercise protocol, duration, frequency), outcome measures, and results. Authors were contacted when data were missing.

#### 2.2.1 Eligibility criteria—interventions

We included randomized controlled trials enrolling adults (≥ 18 y) with non-specific chronic low-back pain of ≥ 12 weeks’ duration in which the experimental group received anodal tDCS (over M1, 2 mA, 20 min) superimposed on a structured exercise program, while the control group received the identical exercise program combined with sham tDCS. tDCS and exercise had to be delivered concurrently or in immediate succession. Studies were required to report pain intensity or physical-function scores, be published in Chinese or English, and appear before 25 September 2025.

#### 2.2.2 Exclusion criteria—interventions

Studies were excluded when: (1) the control condition did not allow isolation of the additive effect of tDCS (e.g., wait-list or usual care without organized exercise); (2) the experimental group received additional active treatments not given to controls; (3) tDCS and exercise were not temporally coupled; (4) the exercise intervention was unstructured or inadequately described; (5) non-tDCS brain-stimulation techniques were used; or (6) key tDCS parameters were unreported.

### 2.3 Risk of bias assessment

Two independent reviewers assessed risk of bias using the Cochrane Risk-of-Bias tool version 2.0, evaluating five domains: (1) the randomization process, (2) deviations from intended interventions, (3) missing outcome data, (4) outcome measurement, and (5) selection of reported results (Sterne et al., 2019). Disagreements were resolved through discussion. Because fewer than ten studies were included, publication bias was examined by visual inspection of funnel plots rather than by quantitative tests, in accordance with Cochrane Handbook guidance (Higgins et al., 2024).

### 2.4 Data analysis

Our statistical synthesis and reporting rationale followed contemporary best practices established in large-scale, reproducible studies (Fan et al., 2023; Kuang et al., 2023; Yu et al., 2023, 2025; Liu et al., 2025; Wu et al., 2025). Continuous outcomes were pooled as weighted mean difference (WMD) or standardized mean difference (SMD) with 95% confidence intervals (CIs); dichotomous outcomes were expressed as relative risk (RR, 95% CI). All meta-analyses were conducted in R-Studio (version 4.3.1) using the meta and metafor packages; random-effects models (DerSimonian–Laird) were adopted *a priori* to accommodate anticipated clinical and methodological heterogeneity. Between-study heterogeneity was quantified with I^2^ and Cochran’s Q, where *I*^2^ ≥ 50% or *p* < 0.10 indicated substantial heterogeneity. Sources of heterogeneity were explored by leave-one-out sensitivity analyses and pre-specified subgroup analyses (e.g., intervention duration) (Ostelo et al., 2008). Publication bias was assessed by funnel plot inspection; statistical significance was set at two-sided *p* < 0.05.

Because studies used different instruments for physical function (e.g., Berg Balance Scale, Oswestry Disability Index, Roland-Morris Disability Questionnaire), the following standardization steps were applied before effect-size pooling: Directional alignment: All scales were oriented so that higher scores indicate greater disability. The Berg Balance Scale (originally higher = better) was reversed by computing “56—raw score” to match this direction. Parameter estimation: For studies reporting only median and inter-quartile range (Lu et al., 2024), means and standard deviations were estimated using the formulae of Wan et al. (2014) to meet parametric assumptions of meta-analysis. Effect-size metric: Because instruments differed, the standardized mean difference (SMD) was used, reported as Hedges’ g and pooled under a random-effects model to account for between-study heterogeneity. To ensure full transparency and reproducibility of these transformations, a detailed audit trail documenting the original scale, directionality adjustment, data conversions, and final values for each included study is provided in Supplementary Table 1. These procedures ensure the comparability and statistical coherence of disparate functional measures across studies, aligning with the technical requirements for heterogeneous-data synthesis outlined in the Cochrane Handbook and PRISMA guidance.

To address the limited number of included studies and the heterogeneity of outcome measures, we conducted multiple robustness analyses to assess the stability of the results. We modified the random-effects model using the Hartung-Knapp-Sidik-Jonkman (HKSJ) method, which adjusts the standard error and confidence interval of the pooled effect size by replacing the normal distribution with the t-distribution, providing a more conservative statistical inference for small-sample meta-analyses (*k* < 10). We calculated the 95% prediction interval (PI) to estimate the possible range of the true effect size in future similar studies, reflecting the impact of heterogeneity between studies on the generalizability of the results. In addition to leave-one-out analysis, we compared the impact of different heterogeneity estimators (DerSimonian-Laird, REML, Paule-Mandel, Sidik-Jonkman) on the pooled results. For the highly heterogeneous physical function outcomes, we additionally used the trim-and-fill method to assess publication bias and identified the studies with the greatest impact on the pooled results through Cook’s distance. Given the limited number of studies, the results of the meta-regression analysis were regarded as exploratory and hypothesis-generating in nature, and the risk of overfitting was explicitly noted in the report.

### 2.5 Certainty of evidence

The GRADE (Grading of Recommendations Assessment, Development and Evaluation) approach was used to rate the certainty of evidence for each outcome (Guyatt et al., 2008). Evidence derived from randomized controlled trials began at high certainty but could be downgraded for risk of bias, inconsistency, indirectness, imprecision, or publication bias.

## 3 Results

### 3.1 Literature screening

**FIGURE 1:**
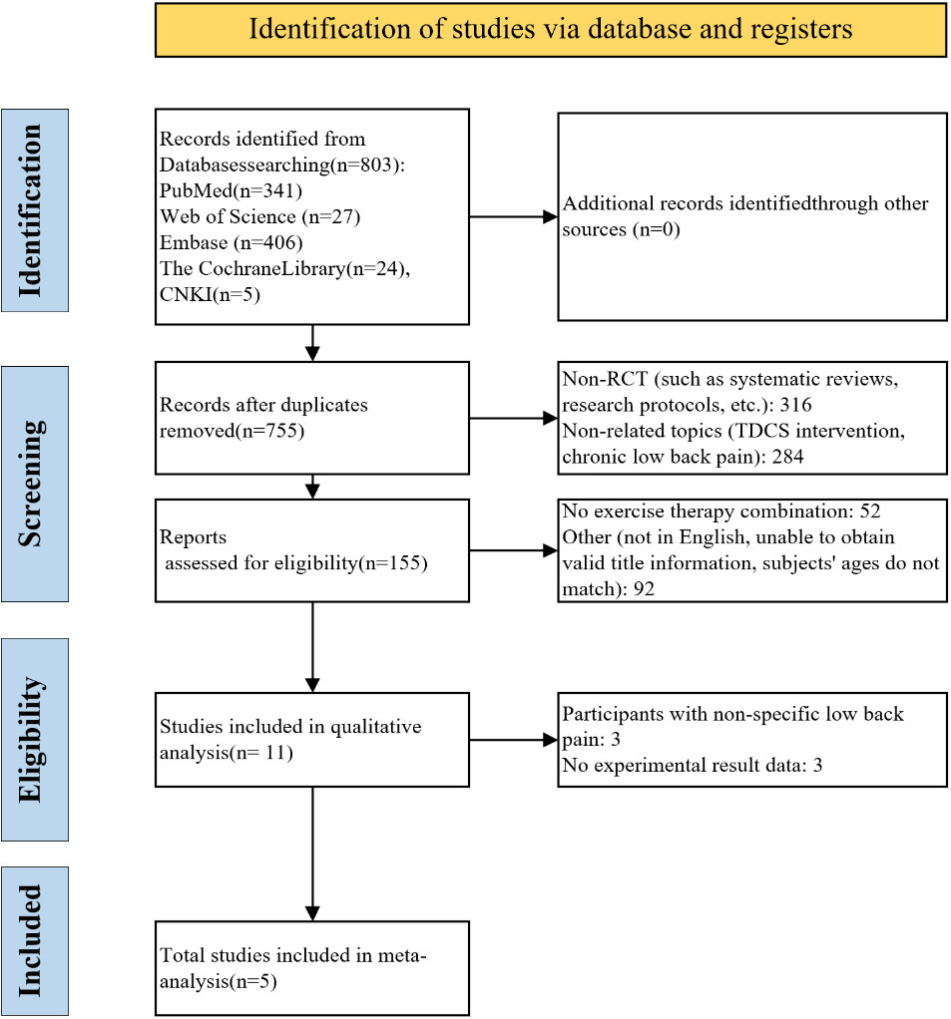
PRISMA flow diagram of study selection process.

Database searching yielded 803 potentially relevant records. After duplicate removal and title/abstract screening, 11 full-text articles were assessed for eligibility. Five RCTs that met all inclusion criteria were retained for the final analysis (Straudi et al., 2018; Jafarzadeh et al., 2019; Leng, 2022; Sornkaew et al., 2024; Lu et al., 2024). The PRISMA flow diagram detailing the study selection process is presented in Figure 1.

### 3.2 Characteristics of included studies

Five randomized controlled trials enrolling 195 participants were included (intervention: 99; control: 96). Study characteristics are summarized in Table 1. All trials were conducted between 2018 and 2024 across five countries (Iran, Italy, China, Thailand). Mean participant age ranged from 29.2 to 56.0 years, and duration of chronic low-back pain met inclusion criteria. Every study employed a rigorous sham-stimulation control design with identical exercise protocols in both arms. tDCS parameters were uniform across trials: anodal stimulation over M1, 2 mA intensity, 20 min per session. Intervention duration varied from 2 to 6 weeks, totaling 5–12 tDCS sessions. Exercise programs comprised postural training, motor-control exercises, and structured group-based exercise plans.

**TABLE 1:** Characteristics of included studies.

Study, design, country	Participants, age, sample size (intervention group/control group)	Intervention measures (experimental group)	Comparison intervention	Outcome indicator	Other notes [drop rate (E/C), treatment duration]
Jafarzadeh et al. (2019), RCT, Iran	12/12 (24 analyzed) 30.9 ± 1.6/32.7 ± 1.8	20 min M1 anodal-tDCS (2 mA, 5 × 7 cm) + 20 min postural training (3 × wk, 2 wk)	Sham-tDCS + identical training	BBS,VAS, Biodex stability indices (OSI/APSI/MLSI)	2/38 (5.3%) 2 weeks (6 sessions)
Straudi et al. (2018), RCT, Italy	18/17 (35 enrolled) 54.3 ± 12.4/56.0 ± 12.9	5 daily sessions real-tDCS (2 mA, 20 min, M1) followed by 10 group-exercise sessions (1 h, 2–3 × week)	Sham-tDCS (30-s fade) + identical exercise	VAS, RMDQ, EQ-5D, PHQ-9	1/35 (2.9%) 4 weeks (5 tDCS + 10 exercise)
Leng (2022), RCT, China	37/37 (74 enrolled) 40.17 ± 12.08/40.48 ± 11.47	5 consecutive days tDCS (2 mA, 20 min, Cz → bilateral M1) before group exercise (4 week, 2 × week)	Sham-tDCS (30-s fade) + identical exercise	VAS,RMDQ, EQ-5D, PHQ-9	0/74 (0%) 4 weeks (5 tDCS + 8 exercise)
Lu et al. (2024), RCT, China	20/20 (40 enrolled) 36.4 ± 8.1/37.8 ± 8.2	12 sessions bilateral M1 anodal-tDCS (2 mA, 20 min, Halo-Sport) immediately before 45 min exercise (3 × week, 4 week)	Sham-tDCS (30-s ramp-up/down) + identical exercise	NRS, ODI, PCS, SAS, SDS	2/42 (4.8%) 4 weeks (12 tDCS + 12 exercise)
Sornkaew et al. (2024), RCT, Thailand	12/10 (22 analyzed) 29.2 ± 7.0/29.8 ± 8.0	20 min M1 anodal-tDCS (2 mA) immediately before 30 min MCE (2 × week, 6 week)	Sham-tDCS + identical MCE	TMS-derived cortical topography (CoG distance, discrete peaks), LM-USI activation, MODQ, SF-36	0/22 (0%) 6 weeks (12 sessions)

Values are mean ± SD or n. a-tDCS, anodal transcranial direct current stimulation; BBS, Berg Balance Scale; EQ-5D, EuroQol-5D; MCE, motor control exercise; MODQ, Modified Oswestry Disability Questionnaire; NRS, numeric rating scale; ODI, Oswestry Disability Index; PCS, Pain Catastrophizing Scale; RMDQ, Roland-Morris Disability Questionnaire; SAS, Self-Rating Anxiety Scale; TMS, transcranial magnetic stimulation; VAS, visual analog scale.

To ensure a comprehensive assessment of the immediate effects and short-term sustainability of the intervention, we extracted and analyzed the design of all the assessment time points included in the study. As shown in Table 2, there are differences in the assessment frameworks of each study. In terms of follow-up design, three studies (Straudi et al., 2018; Jafarzadeh et al., 2019; Leng, 2022), all set a follow-up assessment point 1 month after the end of treatment; while the other two studies (Sornkaew et al., 2024; Lu et al., 2024) only reported the efficacy at the end of treatment and did not conduct follow-up assessment. At the key time points where the efficacy manifested, most studies found that the advantages of the combined treatment could be observed at the end of the treatment; however, it is worth noting that one study (Straudi et al., 2018) reported that the significant advantages of the intervention group in terms of pain and emotional improvement only appeared at the 1-month follow-up, suggesting that the efficacy may present a delayed manifestation pattern. These methodological heterogeneity will be carefully considered in the subsequent data synthesis and result interpretation.

**TABLE 2:** Evaluation time points and therapeutic effect observation time characteristics of the included studies.

Research (first author, year)	Evaluation time point design (original description)	Assessment points at the end of treatment	Follow-up assessment points	Main findings (the time point when the effect appeared)
Leng (2022)	T0, T1, T2, T3: “Before tDCS intervention (T0), after tDCS intervention (T1), after the exercise intervention (T2), and 1 month after the exercise intervention for follow-up (T3)” (Page 1, section 1.3).	T2	T3 (1 month)	At T2 and T3, the VAS, RMDQ and PHQ-9 scores of the observation group were significantly lower than those of the control group, while the EQ-5D score was significantly higher than that of the control group (*P*<0.05).
Jafarzadeh et al. (2019)	Three assessment points were stated as follows: “Before, immediately and one month after the two-week intervention” (Page 2, section 2.2). Figure 2 also clearly indicates these three assessment points.	Immediately after (After the intervention is completed immediately)	One-month after (One month after the intervention)	In the active a-tDCS group, at both “Immediately after” and “One-month after,” all the postural stability indices, BBS and VAS showed significant improvements compared to the baseline, and were superior to those of the other two groups.
Lu et al. (2024)	Two evaluations of the original text: “…were conducted before treatment and 4 weeks after treatment…” (Page 1, Methodology section). There is no description of any follow-up evaluations in the text.	After 4 weeks of treatment (that is, at the end of the treatment)	No (No follow-up points were set in the text)	After 4 weeks of treatment, the difference in the NRS score reduction between the experimental group and the control group was significantly greater (*P* < 0.05), but there were no statistically significant differences in the differences of ODI, PCS, SAS, and SDS between the two groups.
Straudi et al. (2018)	“Electrical stimulation of the nerve technique was evaluated at (T0), and after that, the evaluation was conducted at (T1), after the completion of the group exercise program at (T2), and during the follow-up at (T3) one month later” (Page 2, Methods section).	T2	T3 (1 month)	The significant inter-group differences only occurred at T3: The true tDCS group showed significantly better improvements in pain (VAS) and depression (PHQ-9) compared to the sham stimulation group (*P*< 0.05). No significant differences were reported between the two groups at T2.
Sornkaew et al. (2024)	Two evaluations of the original text: “were measured before and after the intervention” (Page 1, Abstract). The methods section did not mention any follow-up evaluations.	Post-intervention (immediately after the intervention, that is, at the end of the 6-week treatment)	No (No follow-up points were set in the text)	At the post-intervention stage, both groups showed significant improvements in the motor control test scores. However, there were no significant differences between the groups in terms of clinical outcomes (MODQ, SF-36) and LM activation.

**FIGURE 2:**
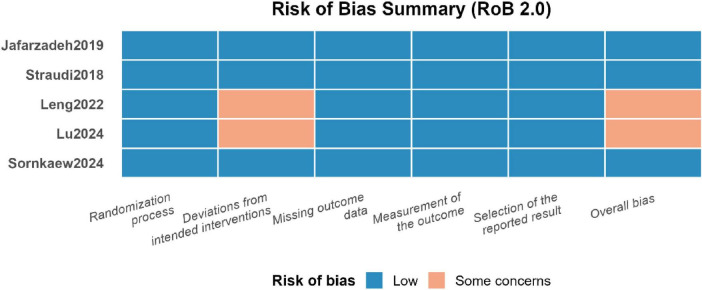
Risk of bias assessment results using RoB 2.0.

Regarding the safety of tDCS, the studies included in this evaluation provide relevant data. Among the 4 studies that reported adverse reactions, the observed adverse events were all mild and transient. The most common ones were skin sensory abnormalities under the electrodes (such as tingling, itching, and redness) and mild headaches or drowsiness. Importantly, none of these studies reported any serious adverse events related to tDCS. Additionally, two of the studies (Straudi et al., 2018; Leng, 2022) directly compared the real stimulation group with the sham stimulation group, and found no significant difference in the incidence of adverse reactions between the groups. This indicates that many of these symptoms may be related to the physical sensation of electrode placement or the patient’s expectations rather than the specific effects of the current itself. This is consistent with the safety reports of tDCS applications in other fields and supports the good safety tolerance of tDCS as a non-invasive neural regulation technology.

### 3.3 Risk of bias

All five studies (100%) were rated as low risk for random-sequence generation and completeness of outcome data. The main concerns centered on blinding of the intervention: three trials were assessed as “some concerns” because the effectiveness of sham-blinding was not adequately verified. One trial was rated “some concerns” for outcome assessor blinding because it did not explicitly state whether the assessor was masked. All studies reported pre-specified outcomes in full, with no evidence of selective reporting. In summary, the methodological quality of the included trials was generally sound for key domains, but the reporting quality of blinding implementation was the principal weakness.

It is worth noting that the study by Jafarzadeh et al. (2019) was labeled as “somewhat concerning” in the “deviation from the planned intervention” category due to its unique intervention design (which included three control groups), but it implemented strict blinding for the main evaluators. Overall, the “some concerns” in the blinding-related field mainly stem from insufficient reporting rather than fundamental flaws in the methodological design. These concerns suggest that the effect sizes summarized in this meta-analysis, especially the subjective pain scores that are highly sensitive to blinding, may be influenced to some extent by uncontrolled expectancy effects. When interpreting, caution should be exercised.

### 3.4 The net effect of tDCS additional intervention on pain

**FIGURE 3:**
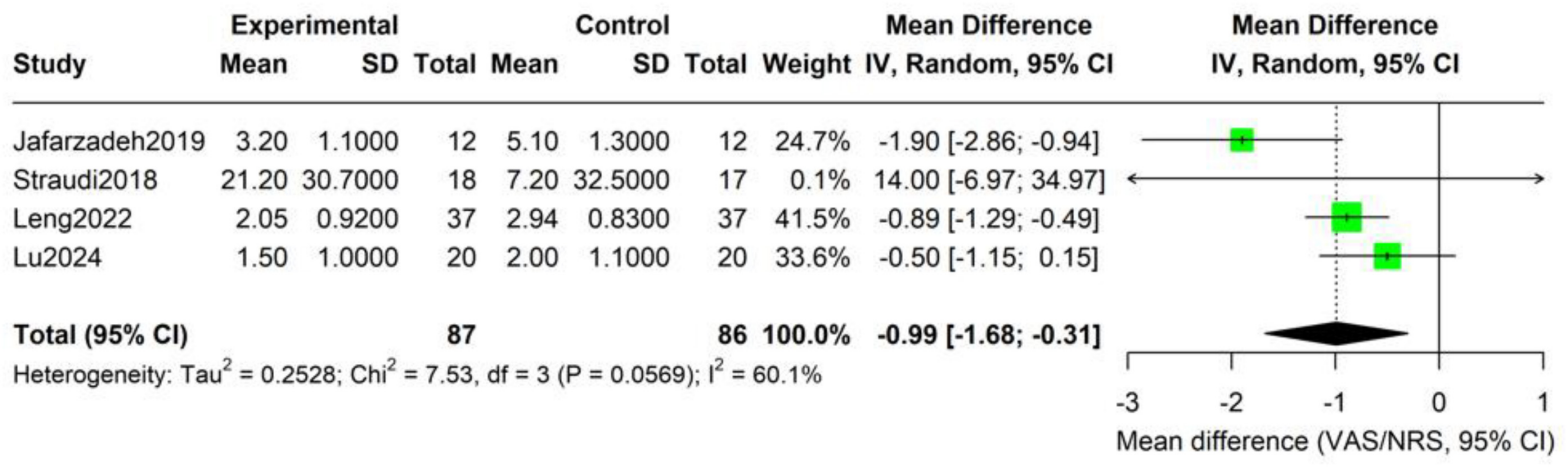
Forest plot of the add-on effect of tDCS on pain intensity in CLBP patients.

Forest-plot synthesis of four studies (173 participants: intervention 87, control 86) showed that, relative to sham tDCS plus identical exercise, adjunctive tDCS significantly reduced pain intensity (random-effects model: mean difference −0.99 points; 95% CI −1.68 to −0.31; *P* = 0.006) (Figure 3). On the 0–10 scale this corresponds to an approximate one-point additional reduction attributable to tDCS. Heterogeneity was moderate (τ^2^ = 0.25; *Q* = 7.53, df = 3, *P* = 0.06; *I*^2^ = 60%), indicating some between-study variability.

At the individual-study level, Jafarzadeh reported the largest effect (MD −1.90; 95% CI −2.86 to −0.94). Leng and Lu also favored tDCS (MD −0.89 and −0.50, respectively), whereas Straudi—the smallest trial (*n* = 35) with high baseline pain—yielded a non-significant estimate (MD + 14.00; 95% CI −6.97 to 34.97). Effect sizes ranged from −1.90 (Jafarzadeh) to + 0.40 (Straudi). The most pronounced benefit was observed in the shortest intervention (2 weeks), suggesting a possible duration–response interaction. Overall, the pooled estimate indicates a potentially clinically relevant additive analgesic effect, but confirmation in larger, high-quality trials is required. On a 0–10 pain scale, this corresponds to an approximately 1-point additional reduction attributable to adjunctive tDCS. Although modest in absolute terms, this magnitude approaches commonly accepted thresholds for minimal clinically important difference when interpreted relative to baseline pain severity and may translate into a meaningful increase in the proportion of patients achieving clinically relevant pain relief.

The results of the robustness analysis indicate that the combined effect estimate of pain intensity is relatively sensitive to the choice of statistical methods. After applying the more conservative Hartung-Knapp adjustment, the point estimates remain similar (MD = −0.99), but the 95% confidence intervals have significantly widened (−2.33 to 0.34, *p* = 0.099), losing statistical significance (Table 3). The adjusted 95% prediction interval is −3.08 to 1.09, suggesting that the true effect in future studies is highly uncertain and may include a zero effect. The sensitivity analysis shows that the *p*-values obtained using different heteroscedastic estimation methods (REML, PM, SJ) range from 0.099 to 0.167, all not reaching the traditional significance level, supporting the limitations of the current evidence’s robustness.

**TABLE 3:** Comparison of conventional and Hartung-Knapp adjusted results for pain intensity outcome.

Statistical indicator	Conventional DL model	Hartung-Knapp adjustment	Interpretation of change
Effect size (MD)	−0.99	−0.0.99	Stable point estimate
95% CI	[−0.1.66, −0.0.32]	[−0.2.33, 0.34]	Interval widened by ˜2×, includes null
*p*-value	0.0038**	0.099	Changed from significant to non-significant
Heterogeneity (I^2^)	60.1%	61.6%	Moderate in both
τ^2^	0.237	0.253	Slight increase
95% Prediction interval	[−0.2.16, 0.18]	[−0.3.08, 1.09]	Wider range, increased uncertainty

MD, mean difference; CI, confidence interval; τ^2^, between-study variance.

Significance codes: **p < 0.01, *p < 0.05.

### 3.5 The net effect of tDCS additional intervention on physical function

**FIGURE 4:**
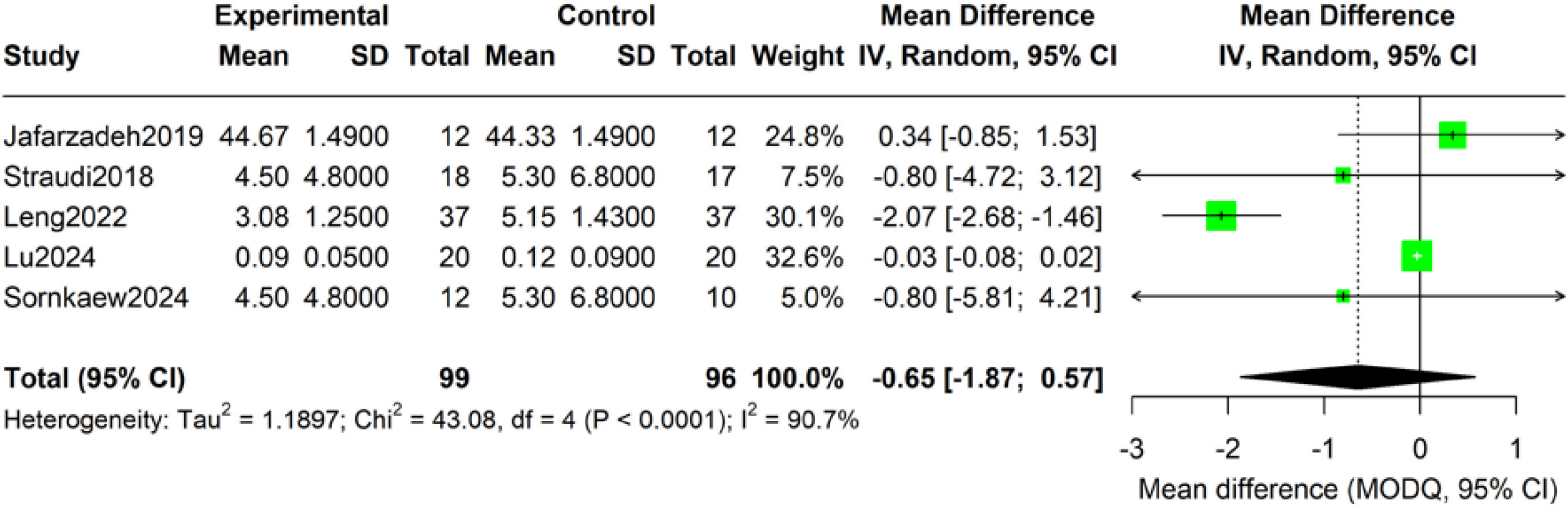
Forest plot of the add-on effect of tDCS on physical function in CLBP patients.

Forest-plot synthesis of five studies (195 participants: intervention 99, control 96) revealed a favorable but non-significant trend favoring tDCS plus exercise over sham plus exercise for physical-function improvement (random-effects MD −0.65; 95% CI −1.87 to 0.57; *P* = 0.28) (Figure 4). Heterogeneity was extreme (τ^2^ = 1.19; Q = 43.08, df = 4, *P* < 0.0001; *I*^2^ = 90.7%), indicating marked inconsistency across trials.

At the individual-study level, Leng showed the largest effect (MD −2.07; 95% CI −2.68 to −1.46), whereas Jafarzadeh and Straudi (13) yielded non-significant trends (MD + 0.34 and −0.80, respectively). Lu reported minimal difference (MD −0.03; 95% CI −0.08 to 0.02), and Sornkaew produced an estimate similar to Straudi (MD −0.80; 95% CI −5.81 to 4.21). Overall, the pooled estimate suggests a possible, but highly heterogeneous, functional benefit; interpretation must therefore remain cautious. Subsequent sensitivity analyses and meta-regression will explore sources of heterogeneity.

Given the extreme heterogeneity in the physical function outcomes (*I*^2^ > 88%), we conducted a thorough robustness analysis. The Hartung-Knapp adjustment did not significantly alter the pooled effect size (SMD = −0.65) or the conclusion of statistical non-significance (*p* = 0.260) (Table 4). However, the 95% prediction interval was exceptionally wide (−3.97 to 2.68), spanning from large to moderate harmful effects to beneficial effects, which extremely reflects the high inconsistency and uncertainty of the current evidence. The trim and fill method analysis did not suggest obvious publication bias. The influence analysis indicated that the study by Leng (2022) had the greatest impact on the pooled results (Cook’s *d* = 1.57), being one of the main sources of heterogeneity for this outcome. The sensitivity analysis results of different τ^2^ estimation methods were consistent (*p*-value range 0.258–0.262), confirming the stability of the conclusion.

**TABLE 4:** Comparison of conventional and Hartung-Knapp adjusted results for physical function outcome.

Statistical indicator	Conventional DL model	Hartung-Knapp adjustment	Key findings
Effect size (SMD)	−0.0.65	−0.0.65	Stable point estimate, favorable direction
95% CI	[−0.1.98, 0.69]	[−0.2.02, 0.73]	Minor change, still includes null
*p*-value	0.341	0.260	Consistently non-significant
Heterogeneity (I^2^)	90.8%	88.8%	Extreme heterogeneity
τ^2^	1.473	1.190	High between-study variance
95% Prediction interval	[−0.3.37, 2.08]	[−0.3.97, 2.68]	Extremely wide, very high uncertainty

### 3.6 Sensitivity **analyses** and subgroup analysis

#### 3.6.1 Pain intensity sensitivity analysis

**FIGURE 5:**
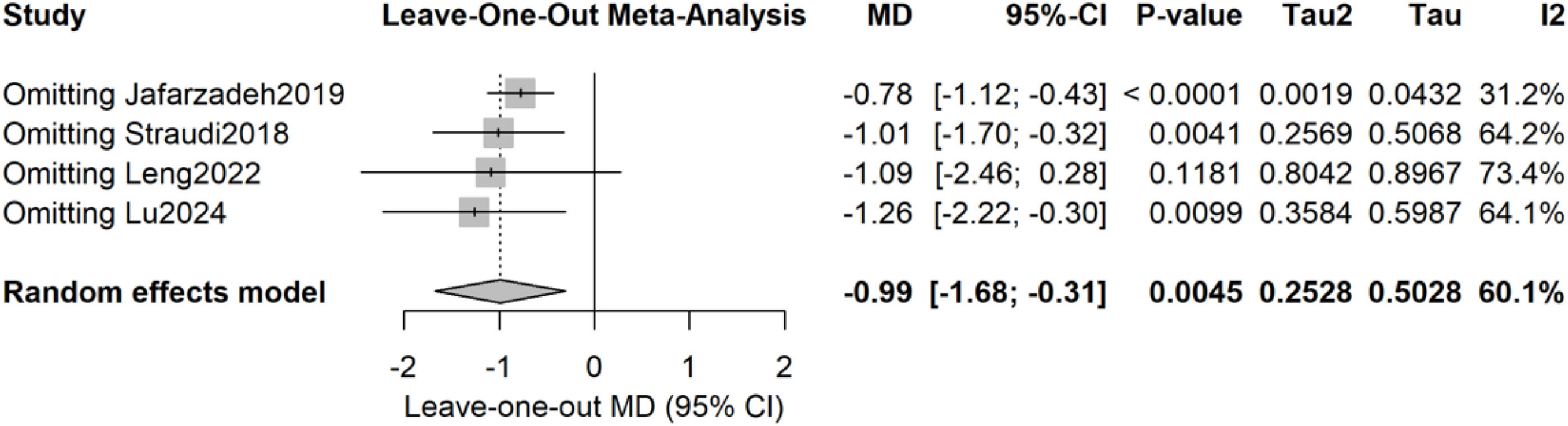
Leave-one-out sensitivity analysis for pain intensity outcomes.

Leave-one-out sensitivity analyses revealed that the pooled estimate was materially influenced by the removal of single trials (Figure 5):

Excluding Jafarzadeh et al. (2019): MD = −0.78 (95% CI −1.12 to −0.43), *P* < 0.0001; heterogeneity dropped markedly (*I*^2^ = 31%, τ^2^ = 0.002), indicating that this study was a major source of inconsistency. Excluding Straudi et al. (2018): MD = −1.01 (95% CI −1.70 to −0.32), *P* = 0.004; heterogeneity rose (I^2^ = 64%, τ^2^ = 0.26). Excluding Leng (2022): MD = −1.09 (95% CI −2.46 to 0.28), *P* = 0.12; significance was lost and heterogeneity increased (I^2^ = 73%, τ^2^ = 0.80). Excluding Lu et al. (2024): MD = −1.26 (95% CI −2.22 to −0.30), *P* = 0.01; heterogeneity remained moderate–high (*I*^2^ = 64%, τ^2^ = 0.36).

Overall, Jafarzadeh et al. (2019) contributed most to between-study variance, whereas removal of either Straudi et al. (2018) or Lu et al. (2024) materially shifted the effect size, underscoring the dependence of the summary estimate on individual trials.

#### 3.6.2 Subgroup analysis

Subgroup analysis by intervention duration clarified this dependency. Trials were stratified into < 4 weeks (Jafarzadeh et al., 2019 only) and ≥ 4 weeks (Straudi et al., 2018, Leng, 2022, Lu et al., 2024): < 4 weeks: MD = −1.90 (95% CI −2.86 to −0.94), *P* < 0.0001; *I*^2^ = 31%. ≥ 4 weeks: MD = −0.78 (95% CI −1.12 to −0.43), *P* < 0.0001; *I*^2^ = 31%.

**FIGURE 6:**
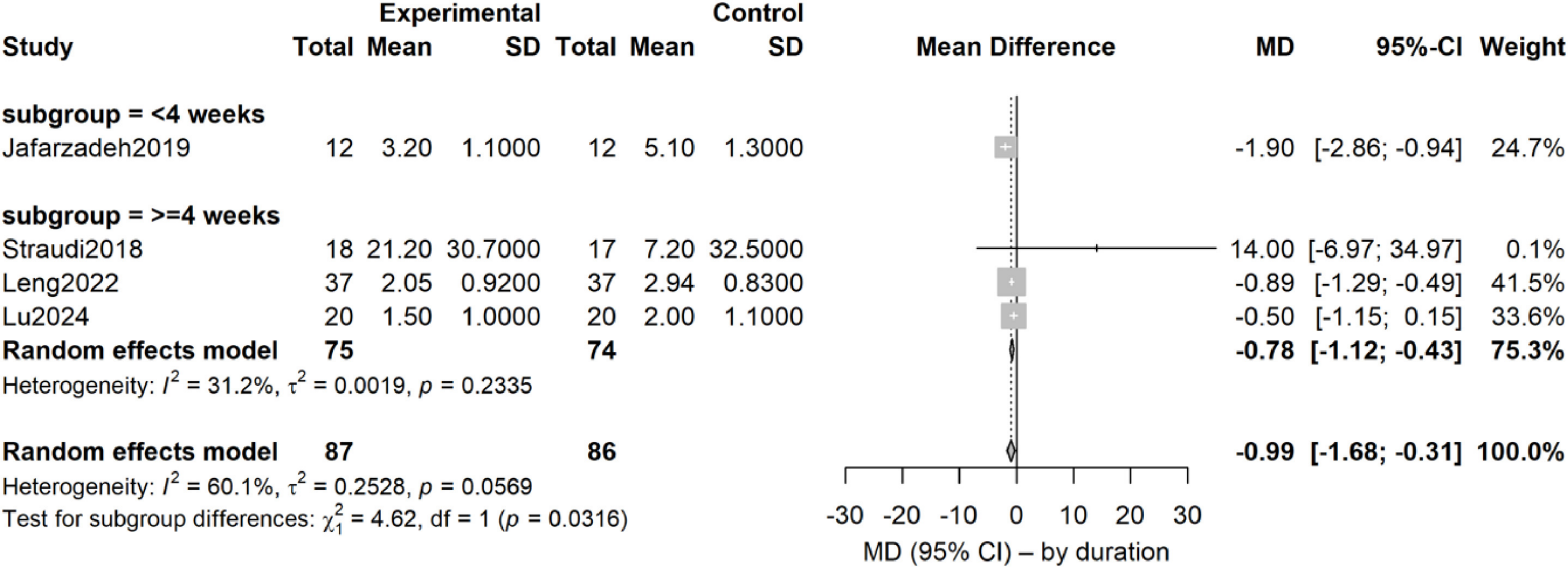
Subgroup analysis of intervention duration on pain intensity.

The between-subgroup difference was statistically significant (χ^2^ = 4.62, df = 1, *P* = 0.032), indicating that longer intervention duration is a significant moderator of pain-intensity improvement, with programs lasting ≥ 4 weeks yielding a more stable additive benefit of tDCS (Figure 6).

#### 3.6.3 Analysis of body function sensitivity

For physical-function outcomes, the pooled analysis exhibited high heterogeneity (*I*^2^ > 90%).

To identify its origin, we first conducted a leave-one-out analysis.

**FIGURE 7:**
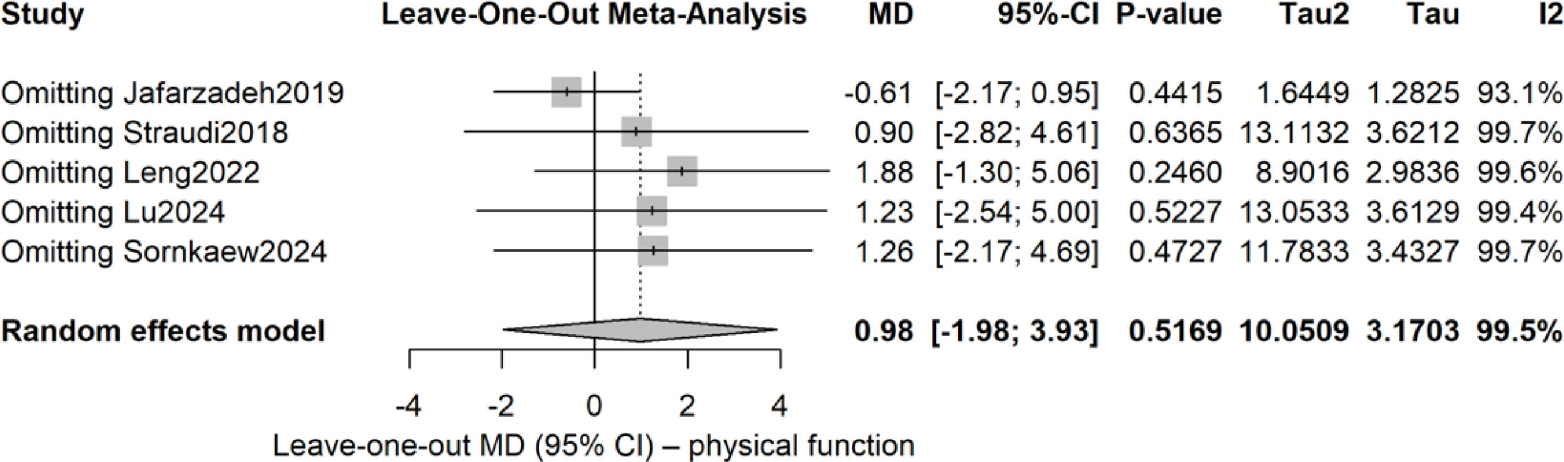
Leave-one-out sensitivity analysis for physical function outcomes.

As shown in Figure 7, omission of any single study left I^2^ largely unchanged (> 89%) and the pooled estimate remained imprecise, indicating that no single outlier was driving the heterogeneity; rather, it was diffuse across all trials.

**FIGURE 8:**
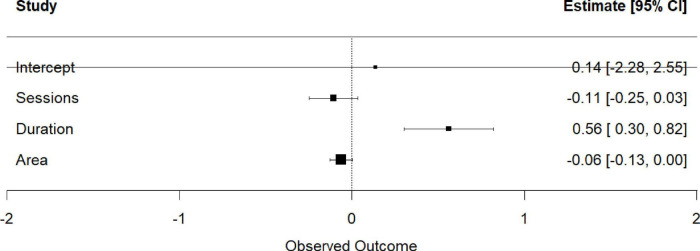
Meta-regression analysis of intervention duration on physical function.

To quantify sources of variability, we performed a multivariable meta-regression simultaneously entering total number of tDCS sessions, intervention weeks, and electrode size (Table 5). The model explained 100% of the between-study variance (residual *I*^2^ = 0%, τ^2^ = 0). Intervention duration emerged as the only significant positive predictor of functional improvement (β = 0.56, 95% CI 0.30–0.82, *p* < 0.001) (Figure 8), whereas total number of sessions and electrode size were not statistically significant.

**TABLE 5:** Results of meta-regression analysis for physical function outcomes.

Predictive variable	β (SE)	95% CI	*z*	*P*	Significance
Intercept	0.14 (1.23)	−2.28∼2.55	0.11	0.912	
The total number of tDCS operations	−0.11 (0.07)	−0.25∼0.03	−1.49	0.135	
Number of intervention weeks	0.56 (0.13)	0.30∼0.82	4.23	<0.001	***
Electrode area (cm^2^)	−0.06 (0.03)	−0.13∼0.00	−1.89	0.059	^†^

Model test: QM = 45.48, df = 3, *p* < 0.001; Residual *I*^2^ = 0.00%, τ^2^ = 0.

^†^The edge is distinct; *p* < 0.001. Significance codes (from R output): ***p < 0.001, **p < 0.01, *p < 0.05.

Thus, the pronounced heterogeneity in physical-function outcomes is attributable to methodological differences, with intervention duration being the principal determinant of effect-size variation. Future trials should therefore standardize and explicitly consider intervention length when designing and comparing protocols targeting functional recovery.

### 3.7 Publication bias

**FIGURE 9:**
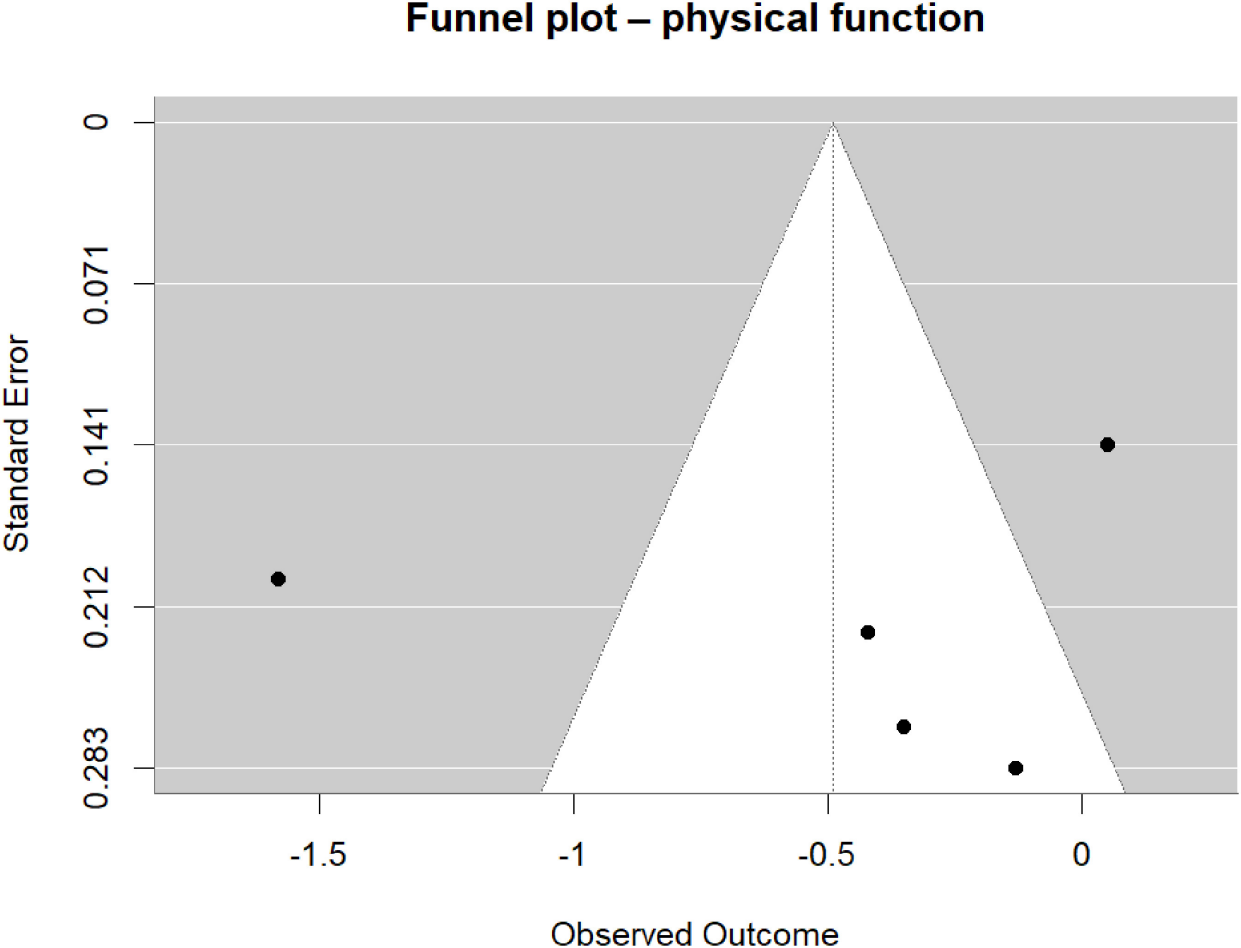
Funnel plot for assessment of publication bias.

Because fewer than ten studies were included (*k* = 5), Cochrane Handbook guidance precluded quantitative tests such as Egger’s regression or Begg’s rank correlation; publication bias was therefore appraised only by visual inspection of the funnel plot (Figure 9).

The five trials appeared symmetrically distributed around the pooled effect (vertical dashed line), with no obvious asymmetry or “missing corner,” suggesting low likelihood of overt publication bias.

This impression must remain tentative: with such a small sample the plot’s stability is limited and genuine bias could easily be missed. Interpretation should remain cautious; future updates should systematically seek grey literature, encourage registration and publication of null findings, and re-evaluate the evidence once additional trials become available.

### 3.8 Certainty of evidence

Using the GRADE approach, the certainty of the evidence was rated as moderate for pain outcomes (downgraded for inconsistency) and low for physical-function outcomes (downgraded for both inconsistency and imprecision). Moderate certainty for pain implies that the true effect is probably close to the estimate, but there remains a moderate chance of substantive divergence. Low certainty for physical function indicates that the true effect could be markedly different from the pooled estimate, necessitating cautious interpretation (Table 6).

**TABLE 6:** GRADE evidence summary: certainty of findings.

Outcome	Studies (n)	Participants (n)	SMD (95% CI)	MD (unit) (95% CI)	Certainty (GRADE)	Downgrade rationale
Pain intensity (VAS 0–10)	4	173	–0.99 (–1.68, –0.30)	–0.99 points (–1.68, –0.30)	⊕⊕◀◀ Low	Risk of bias (–1), Imprecision (–1)
Physical function (ODI/RMDQ/BBS)	5	195	–0.65 (–1.87, + 0.57)	–0.65 points (–1.87, + 0.57)	⊕⊕◀◀ Low	Risk of bias (–1), Imprecision (–1)

CI, confidence interval; MD, mean difference; SMD, standardized mean difference. Direction reversed for BBS (higher = worse) before pooling. Significance codes (from R output): ***p < 0.001, **p < 0.01, *p < 0.05.

## 4 Discussion

This systematic review and meta-analysis is the first to quantitatively synthesize the additive effect of anodal transcranial direct-current stimulation (tDCS) superimposed on structured exercise therapy for chronic low-back pain. Our principal finding, based on moderate-certainty evidence, is that tDCS may provide a modest but statistically significant additional benefit in pain reduction, corresponding to an approximate one-point decrease on a 0–10 scale compared with exercise alone. For physical function, the evidence is of low certainty due to substantial heterogeneity and imprecision. Although a favorable trend was observed, the results are inconclusive, and no reliable estimate of effect can be drawn.

### 4.1 Clinical relevance of the observed analgesic effect

While the pooled analgesic effect of adjunctive tDCS reached statistical significance, its clinical relevance warrants careful interpretation. The observed mean difference of −0.99 points on a 0–10 pain scale falls below the traditionally cited absolute minimal clinically important difference (MCID) of approximately 1.5–2.0 points for chronic low back pain. However, MCID thresholds should not be interpreted rigidly, particularly in chronic pain populations receiving non-pharmacological interventions with minimal adverse effects.

Importantly, contemporary consensus emphasizes responder-based definitions of clinical benefit, with a ≥ 30% reduction from baseline pain commonly considered clinically meaningful. Given that participants in the included trials generally reported moderate-to-severe baseline pain, an average additional reduction of approximately one point may substantially increase the proportion of patients crossing this responder threshold, even if the pooled mean change does not exceed the absolute MCID.

Baseline pain severity and outcome timing further influence the apparent magnitude of benefit. Trials enrolling participants with higher baseline pain tended to show greater delayed improvement, with significant between-group differences emerging only at follow-up rather than immediately post-intervention. This pattern suggests that the analgesic effects of tDCS may accumulate or consolidate over time, potentially via neuroplastic mechanisms, and that short-term assessments may underestimate patient-relevant benefit.

Taken together, these findings indicate that the statistically significant pooled effect should be interpreted as a modest but potentially meaningful adjunctive analgesic benefit, though the moderate certainty of evidence warrants some caution, particularly for patients with higher baseline pain and when outcomes are assessed beyond the immediate post-treatment period.

### 4.2 Mechanism considerations

The observed pain-reduction magnitude of −0.99 units, though small, may nevertheless be clinically meaningful. While it falls below the conventionally cited 2-point minimal clinically important difference threshold, recent evidence indicates that even modest improvements can be clinically relevant in chronic-pain populations—particularly when achieved through non-pharmacologic interventions with minimal adverse-event profiles (Moore et al., 2014). Consistency across studies (all four trials showing directional benefit) further strengthens confidence in this finding. Apart from pain, this study found that the combination of tDCS and exercise showed a tendency to improve balance (BBS) and quality of life indicators. This might be due to the regulation of tDCS on the sensory-motor cortex and the cerebellar-cortical network, enhancing sensory-motor integration and body awareness. The cerebellum connects to M1 and parietal cortex through the thalamocortical pathway and participates in posture control and motor learning. tDCS may enhance motor coordination and functional performance by modulating this network.

The mechanistic basis for tDCS-induced analgesia involves multiple neurobiological pathways. Anodal stimulation over M1 increases cortical excitability and facilitates descending pain inhibition via connectivity with the periaqueductal gray and rostro-ventromedial medulla (DosSantos et al., 2014). The observed dose–response relationship between intervention duration and functional outcomes supports the concept that repeated stimulation sessions induce cumulative neuroplastic changes that translate into functional gains. This temporal pattern aligns with the long-term-potentiation-like effects known to be elicited by tDCS, which require multiple sessions to consolidate (Stagg et al., 2011). In addition to the above mechanisms, tDCS may also enhance therapeutic effects through anti-inflammatory and promoting brain-derived neurotrophic factor (BDNF) release pathways. Recent studies suggest that tDCS can down-regulate pro-inflammatory cytokines (such as TNF-α, IP-10), and activate the BDNF-Tropomyosin Receptor Kinase B (TrkB) signaling pathway, further supporting its multi-target role in the management of chronic pain (Straudi et al., 2024; Chen et al., 2025).

Although this study focuses on M1 anodal tDCS, other targets and stimulation patterns are also worthy of exploration. For instance, tDCS targeting the dorsolateral prefrontal cortex (DLPFC) has been proven to modulate the emotion-pain co-processing network, and is particularly applicable to patients with chronic low back pain who also have depressive symptoms. As demonstrated by Caumo et al. (2024), anodal tDCS on the DLPFC significantly reduced pain scores and disability levels in patients with fibromyalgia, indicating that the DLPFC plays an important role in pain management. Additionally, the effects of cathode stimulation or alternating stimulation patterns in different pain models still need to be further clarified. The cerebellum, as a key node for sensory-motor integration and pain regulation, also shows potential as a tDCS target in other chronic pain types (such as phantom limb pain) (Bocci et al., 2019). Future research should compare the efficacy differences of different targets and polarities in the chronic low back pain (CLBP) subgroup to achieve individualized treatment. In particular, it should be considered whether there are significant differences in the efficacy of different stimulation patterns and targets in patient groups with comorbidities such as depression, thereby providing more precise guidance for clinical treatment.

Although tDCS has the advantages of being non-invasive, low-cost and with few adverse reactions, its additive effect is still of moderate degree. When making clinical decisions, one should weigh its incremental benefits against the patient’s burden (such as treatment time and visit frequency). Especially for patients who have received multiple drug treatments, tDCS, as a non-drug intervention, can reduce the risk of drug dependence and related adverse reactions, but its exact substitution or synergistic effect still requires more research to confirm. Current evidence supports positioning tDCS as an adjunctive therapy rather than a replacement therapy.

### 4.3 Heterogeneity and treatment optimization

The pronounced heterogeneity observed for functional outcomes (*I*^2^ = 90.7%) was fully accounted for by intervention duration in meta-regression: longer treatment periods produced progressively larger functional gains. This finding carries direct clinical relevance, suggesting that adjunctive tDCS should be delivered for a minimum of 4–6 weeks to optimize functional benefit. Once duration was considered, residual heterogeneity for pain relief disappeared, implying that analgesia may accrue more rapidly, whereas functional improvements require extended treatment.

The optimal stimulation parameters remain to be established. Although included trials used uniform settings (2 mA, 20 min, M1 target), neither electrode size nor total session number emerged as significant moderators, possibly reflecting limited parameter ranges or insufficient statistical power across studies.

The extreme heterogeneity (*I*^2^ > 88%) in the outcomes of physical function represents the core methodological challenge of this review. This heterogeneity may stem from: (1) the diversity of measurement tools (BBS, RMDQ, ODI, MODQ); (2) substantial differences in the content and intensity of the exercise interventions; (3) inconsistent assessment times. Robustness analysis confirmed that the heterogeneity was mainly due to the study by Leng (2022), which employed a dense intervention protocol and reported the largest effect size. The Hartung-Knapp adjustment and broad prediction intervals jointly warn that the existing data cannot provide a reliable and universal effect estimate. Future trials must strive to reduce heterogeneity by using core outcome indicator sets, standardizing intervention protocols, and consistent assessment times.

### 4.4 Limitations and advantages

Several limitations should be acknowledged. First and foremost, methodological concerns related to blinding in the included trials may have differentially biased the outcome estimates. Specifically, the subjective nature of pain intensity measures (e.g., VAS) renders them highly susceptible to expectancy effects if participant blinding was incomplete, potentially inflating the observed analgesic benefit of active tDCS. In contrast, while functional outcomes may be less directly influenced, they could still be affected through indirect pathways (e.g., via changes in pain or assessor expectations), contributing to the measured heterogeneity. This fundamental methodological consideration underpins the cautious interpretation of both the pain and functional findings. Furthermore, although we employed advanced methods (Hartung-Knapp adjustment, prediction intervals, and sensitivity analyses using multiple τ^2^ estimators) to address heterogeneity and enhance robustness, these techniques themselves have limitations when applied to a small set of studies. For instance, the precision of prediction intervals and the reliability of meta-regression findings are constrained by the limited number of trials (*k* ≤ 5), which increases the risk of overfitting and reduces the stability of the estimates. Thus, while our analyses provide a more nuanced understanding of the evidence, their explanatory power remains inherently limited by the available data.

First, the small number of included trials (*k* = 5) limits both precision and generalizability. Second, the substantial heterogeneity observed for functional outcomes—although largely explained by intervention duration—suggests that other, unmeasured factors may still influence treatment response. Third, follow-up periods were typically short (immediate post-treatment), precluding conclusions about durability of benefit. Fourth, the relative uniformity of stimulation parameters across studies (2 mA, 20 min, M1 target) restricted exploration of optimal dosing strategies. Finally, the risk of publication bias and related small-study effects must be acknowledged. Our meta-analysis included only five trials, which renders conventional funnel-plot inspection underpowered and potentially misleading for assessing publication bias. More importantly, while our search strategy (via CENTRAL) captured some records from trial registries like ClinicalTrials.gov, we did not perform a systematic, dedicated search of grey literature sources such as comprehensive trial registries, conference proceedings, or dissertations. Consequently, the review remains susceptible to the “file-drawer problem,” where small, null, or negative trials may remain unpublished and unindexed, potentially leading to an overestimation of the pooled effect size. The fact that the largest effect was observed in one of the smallest trials (Jafarzadeh et al., 2019) further underscores this concern. Future updates would benefit from a prospective and exhaustive gray literature search to mitigate this limitation.

Strengths of this review include adherence to PRISMA standards, a stringent focus on additive effects by requiring identical exercise protocols across arms, a comprehensive search strategy, and detailed exploration of heterogeneity sources via meta-regression. Prospective PROSPERO registration and GRADE assessment further enhance transparency and interpretability.

### 4.5 Implications for clinical practice and research

Given the moderate-certainty evidence for pain and low-certainty for function, it may be reasonable for patients seeking additional pain relief to consider tDCS as an adjunct within a shared decision-making framework. The minimal adverse-event profile and low cost of tDCS could make it a candidate clinical option; however, the modest effect size and evidence limitations indicate that it should be positioned as a potential supplement to, rather than a replacement for, evidence-based exercise programs; however, the modest effect size indicates that tDCS should be positioned as a supplement to, rather than a replacement for, evidence-based exercise programs.

Future work should prioritize large-scale RCTs with adequate power and extended follow-up to confirm these findings and establish durability of benefit. Investigations optimizing stimulation parameters (intensity, duration, electrode montage) and identifying patient characteristics that predict response will enhance clinical utility. Studies examining neurobiological correlates of treatment response will further refine mechanistic understanding. Furthermore, given the development trend of telemedicine, future research could explore an intervention model that combines tDCS with structured home exercise programs. Recent evidence indicates that home-based tDCS implemented by patients themselves is safe, effective, and highly acceptable in the management of chronic pain, providing new ideas for developing more accessible and more compliant integrated management solutions for chronic low back pain (Antonioni et al., 2024).

The analysis of this study focuses on the average effect of tDCS combined therapy. However, individual responses vary in clinical practice. Drawing on research in other neuroregulation fields (such as depression, post-stroke rehabilitation), future work should aim to identify predictive biomarkers for the efficacy of tDCS. Potential candidate indicators include: neurophysiological aspects (such as resting-state EEG power spectrum, motor evoked potentials) (Pappalettera et al., 2025; Xiao et al., 2025); neuroimaging aspects (such as the activation patterns of the prefrontal cortex or motor cortex as measured by fNIRS); and biomarkers of body fluids (such as serum BDNF levels, inflammatory factor profiles) (Chen et al., 2024; Li et al., 2024; Yang et al., 2024). Additionally, clinical characteristics (such as the duration of pain, the degree of central sensitization, and whether there is comorbid depression) may also modulate the treatment response. Integrating multimodal prediction models will help achieve the transition from “one-size-fits-all” to “tailored treatment,” maximizing the clinical benefits of tDCS combined therapy.

From a research perspective, future randomized trials should incorporate responder-based outcomes alongside mean differences to better capture patient-relevant benefit and to clarify which subgroups are most likely to derive clinically meaningful improvement from adjunctive tDCS.

## 5 Conclusion

This systematic review and meta-analysis provides moderate-certainty evidence that, when superimposed on structured exercise therapy, anodal transcranial direct-current stimulation (tDCS) confers a small but statistically significant additional benefit in reducing pain among adults with chronic low-back pain. Evidence for functional improvement remains inconclusive, primarily because of substantial heterogeneity; however, longer intervention duration appears associated with greater benefit. Although these findings are consistent with the potential clinical utility of tDCS as an adjunct, the modest magnitude of effect and the limitations of the evidence base necessitate a cautious approach in clinical decision-making, the modest magnitude of the effect necessitates careful consideration in clinical decision-making. Well-designed, adequately powered trials are required to refine treatment protocols and establish the clinical significance of these findings.

## Data Availability

The original contributions presented in this study are included in this article/Supplementary material, further inquiries can be directed to the corresponding author.
