# Supplementary material for: Transcranial direct current stimulation combined with exercise therapy for chronic low back pain: a systematic review and meta-analysis
Source: Front Hum Neurosci. 2026 Jan 16;19:1721182. doi: 10.3389/fnhum.2025.1721182 (PMC12857311; doi:10.3389/fnhum.2025.1721182)
Supplement: Supplementary file 1 [file Data_Sheet_1.pdf]

Author(s):
Question: Active tDCS plus exercise therapy compared to sham tDCS plus exercise therapy for adults with non-specific chronic low back pain
Setting:
Bibliography:

Certainty assessment							№ of patients		Effect		Certainty	Importance
**№ of studies**	**Study design**	**Risk of bias**	**Inconsistency**	**Indirectness**	**Imprecision**	**Other considerations**	**active tDCS plus exercise therapy**	**sham tDCS plus exercise therapy**	**Relative (95% CI)**	**Absolute (95% CI)**		
**□□□□□□ (assessed with: VAS 0-10 (0 = no pain, 10 = worst pain))**												
4	randomised trials	serious[a]	serious[b]	not serious	serious[c]		87	86	-	MD **0.99 points lower** (1.68 lower to 0.31 lower)	-[a,b,c]	CRITICAL
**New outcome (assessed with: ODI, RMDQ, BBS (higher score = worse function))**												
5	randomised trials	serious[a]	serious[d]	not serious	serious[e]		99	96	-	MD **0.65 points lower** (1.87 lower to 0.57 higher)	-[a,d,e]	CRITICAL

CI: confidence interval; **MD:** mean difference

Explanations

a. Downgraded for risk of bias (some concerns in multiple domains).
b. Downgraded for substantial heterogeneity ($I^2 > 50\%$).
c. small sample, wide CI.
d. very high heterogeneity ($I^2 = 91\%$).
e. small sample, CI crosses null.
