# Supplementary material for: Transcranial direct current stimulation combined with exercise therapy for chronic low back pain: a systematic review and meta-analysis
Source: Front Hum Neurosci. 2026 Jan 16;19:1721182. doi: 10.3389/fnhum.2025.1721182 (PMC12857311; doi:10.3389/fnhum.2025.1721182)
Supplement: Supplementary file 2 [file Table_1.pdf]

Supplementary Table S1. Audit trail for the standardization of physical function outcomes across included studies.

Study (First Author, Year)	Original Functional Scale Used	Original Scale Direction (Higher Score =)	Transformation Applied for Directional Alignment	Original Reported Statistics (Mean±SD or Median[IQR])	Converted/Final Mean (SD) for Analysis	Notes on Conversion & Assumptions
Jafarzadeh, 2019	Berg Balance Scale (BBS)	Better function	Reversal: Final Score = 56 - Raw BBS Score	Reported as Mean (SD)	Mean: 11.33, SD: 1.49*	*SD remains unchanged after linear reversal. Direction now aligned (higher = worse).
Straudi, 2018	Roland-Morris Disability Questionnaire (RMDQ)	Worse function (higher disability)	None. Higher score already indicates worse function.	Reported as Mean (SD)	Mean: 4.50, SD: 4.80 (Exp.)	Data extracted from forest plot. Scale range: 0-24.
Leng, 2022	Roland-Morris Disability Questionnaire (RMDQ)	Worse function	None.	Reported as Mean (SD)	Mean: 3.08, SD: 1.25 (Exp.)	Scale range: 0-24.
Lu, 2024	Oswestry Disability Index (ODI, %)	Worse function	None for direction. Conversion from median[IQR] to mean(SD).	Reported as Median [IQR]: 8 [6, 12] for experimental group; 12 [8,	Exp. Mean: 9.0, SD: 4.6* Ctrl Mean: 12.0, SD: 6.0*	*Mean and SD estimated using Wan et al. (2014) formulas (see Methods). Assumes approximate normality within IQR.

				16] for control.		
Sornkaew, 2024	Modified Oswestry Disability Index (MODQ, %)	Worse function	None.	Reported as Mean (SD)	Mean: 4.50, SD: 4.80 (Exp.)	Data likely identical to Straudi et al. due to reporting in forest plot; confirmed from study text.

Table Footnote:

BBS: Berg Balance Scale (Range 0-56). **RMDQ**: Roland-Morris Disability Questionnaire (Range 0-24). **ODI/MODQ**: Oswestry (Modified) Disability Index (Range 0-100%). **Exp**.: Experimental group. **Ctrl**: Control group. **IQR**: Interquartile range. **SD**: Standard deviation.
