# Supplementary material for: Transcranial direct current stimulation combined with exercise therapy for chronic low back pain: a systematic review and meta-analysis
Source: Front Hum Neurosci. 2026 Jan 16;19:1721182. doi: 10.3389/fnhum.2025.1721182 (PMC12857311; doi:10.3389/fnhum.2025.1721182)
Supplement: Supplementary file 4 [file Table_3.docx]

Search expression

**PubMed**：

(

("Electric Stimulation Therapy"[Mesh] OR transcranial[tiab] OR tDCS OR "direct current" OR "non-invasive brain stimulation" OR NIBS[tiab])

) AND (

("Exercise"[Mesh] OR exercise*[tiab] OR "physical therapy" OR physiotherapy OR training[tiab] OR "motor rehabilitation")

) AND (

("Back Pain"[Mesh] OR "low back pain" OR "lower back pain" OR "lumbar pain" OR "chronic back" OR CLBP)

) AND (

random*[tiab] OR RCT[tiab] OR "clinical trial"[pt] OR "controlled trial"[pt]

)

**WOS**: TS=((transcranial OR tDCS OR "non-invasive brain stimulation" OR NIBS OR "direct current") AND (exercise* OR training OR physiotherapy OR "physical rehabilitation") AND ("back pain" OR "lumbar pain" OR CLBP) AND (random* OR RCT))

**COCHRANE**

#1 (transcranial OR tdcs OR "non-invasive brain stimulation" OR nibs OR "direct current"):ti,ab,kw

#2 (exercise* OR training OR physiotherapy OR "physical rehabilitation"):ti,ab,kw

#3 ("back pain" OR "lumbar pain" OR clbp):ti,ab,kw

#4 #1 AND #2 AND #3

#5 #4 AND (random* OR rct):ti,ab,kw

**EMBSE**

'electric stimulation therapy'/exp OR transcranial:ti,ab OR tdcs:ti,ab OR 'non-invasive brain stimulation':ti,ab OR nibs:ti,ab OR 'direct current':ti,ab

'exercise'/exp OR exercise*:ti,ab OR training:ti,ab OR physiotherapy:ti,ab OR 'physical rehabilitation':ti,ab

'back pain'/exp OR 'back pain':ti,ab OR 'lumbar pain':ti,ab OR clbp:ti,ab

1 AND 2 AND 3

4 AND (random*:ti,ab OR rct:ti,ab OR 'controlled trial':ti,ab OR 'randomized controlled trial'/exp)

CNKI

(SU='经颅直流电刺激' OR SU='tDCS' OR SU=' transcranial direct current stimulation' OR SU='非侵入性脑刺激') AND (SU='运动' OR SU='锻炼' OR SU='运动疗法' OR SU='物理治疗' OR SU='康复训练') AND (SU='慢性下背痛' OR SU='慢性腰痛' OR SU='慢性非特异性腰痛' OR SU='CLBP') AND (SU='随机' OR SU='RCT' OR SU='随机对照试验')
